# HIV screening among patients seeking care at Xuanwu Hospital: A cross-sectional study in Beijing, China, 2011–2016

**DOI:** 10.1371/journal.pone.0208008

**Published:** 2018-12-17

**Authors:** Rui Li, Guoguang Zhao, Jia Li, Jennifer M. McGoogan, Chu Zhou, Yan Zhao, Zhigang Liang, Haiping Zhang, Ya Zuo, Lan Lan, Zunyou Wu

**Affiliations:** 1 Information Center, Xuanwu Hospital, Capital Medical University, Beijing, China; 2 Medical Department, Xuanwu Hospital, Capital Medical University, Beijing, China; 3 National Center for AIDS/STD Control and Prevention, Chinese Center for Disease Control and Prevention, Beijing, China; 4 Department of Dermatology and Venereology, Xuanwu Hospital, Capital Medical University, Beijing, China; 5 Disease Control Department, Xuanwu Hospital, Capital Medical University, Beijing, China; 6 West China Biomedical Big Data Center, West China Hospital, Sichuan University, Chengdu, China; 7 Department of Epidemiology, Fielding School of Public Health, University of California, Los Angeles, United States of America; Jiangsu provincial Center for Disease Control and Prevention, CHINA

## Abstract

**Objectives:**

One-third of people living with HIV in China are still unaware of their status, so we sought to better understand HIV testing in the general hospital setting in China.

**Methods:**

A cross-sectional study was conducted using the electronic medical records of all patients who attended Xuanwu Hospital in Beijing, January 1, 2011 to December 31, 2016. HIV screening and detection rates and characteristics of patients diagnosed with HIV were assessed.

**Results:**

Overall, 235,961 patients were screened, for a screening rate of 1.4%. Although most were outpatients (98.4%), screening rate was higher among inpatients (70.0% versus 0.4%), and highest in internal medicine (36.1%) and surgery (33.3%) departments. A total of 140 patients were diagnosed with HIV, for a detection rate of 5.93 per 10,000. Detection rates were highest among outpatients (9.34 per 10,000), and patients attending the dermatology and sexually transmitted infection (STI) department (153.85 per 10,000). Most diagnoses were made among males (91.4%), aged 20–39 (67.1%), who reported becoming infected through homosexual contact (70.0%).

**Conclusions:**

HIV screening in China’s general hospitals needs to be improved. More focus should be placed on screening outpatients, especially in the dermatology and STI department, and young men.

## Introduction

HIV testing has been a cornerstone of China’s work toward control and prevention since the beginning of its HIV/AIDS epidemic response[1]. In 2002, fees for HIV voluntary counseling and testing (VCT) were eliminated, and by 2004, coverage had been expanded nationwide[2]. Also in 2004, Henan province became the first to embark on a large-scale testing program—public health workers went door-to-door in search of those who may have donated blood in the 1990s to offer them free HIV screening. This program yielded 23,157 new cases in just 3 months. Mass screening mobilization efforts then took place in other provinces among key populations at high risk of infection through 2005[1]. Mandatory HIV testing (MHT) was implemented at detention centers, re-education/labor camps, and methadone maintenance treatment clinics in 2005[1]. In 2007, HIV provider-initiated testing and counseling (PITC) was introduced and has since expanded dramatically[2]. Over the subsequent 10 years, strong focus on testing has persisted—45 million HIV tests administered nationwide in 2008 rose to nearly 145 million in 2015, 72% of which resulted from PITC in medical settings[1].

Despite all of these efforts, an estimated 276 thousand people living with HIV (PLWH) in China still did not know their status as of the end of 2015[3], and it is off track for meeting the Joint United Nations Programme on HIV/AIDS (UNAIDS) 90-90-90 Targets—90% of PLWH should know their status, 90% of those who have been diagnosed should be on treatment, and 90% of those on treatment should achieve viral suppression[4, 5]. In order to take China from an estimated 68% at the end of 2015[3], to the UNAIDS “First 90” target of 90%, we must better understand how testing is (and is not) working in China today.

According to Chinese Government data, a total of 7.9 billion visits were made to all medical institutions nationwide in 2015, with a considerable proportion absorbed by general hospitals—2.3 billion outpatients and 123 million inpatients attended Chinese general hospitals in 2015[6]. All general hospitals in China offer HIV screening, which visitors may access in three different ways. First, they may attend an in-hospital VCT site. This is typically a separate location or department within the hospital that takes walk-in patients and offers basic screening and counseling. Second, visitors may attend the hospital’s dermatology and sexually transmitted infections (STI) department. There, they may request a test themselves, or a provider may request a test on their behalf (as PITC). Third, PITC may occur in any department. However, HIV testing is required for patients in certain circumstances—those undergoing particular procedures (e.g. surgeries, dialysis, blood transfusions, pregnancy tests), and those diagnosed with specific infections or malignancies associated with HIV infection (e.g. tuberculosis, Kaposi’s sarcoma).

We sought to better understand HIV testing uptake in the Chinese general hospital setting for the purpose of future improvement of testing services. Therefore, we aimed to investigate HIV screening and detection rates (primary objective), and characteristics of patients diagnosed with HIV infection (secondary objective), at Xuanwu Hospital, Capital Medical University (hereafter referred to as Xuanwu Hospital) in downtown Beijing, China from 2011 to 2016.

## Methods

### Design, setting, and data source

A cross-sectional study design was used to investigate HIV screening rates and HIV detection rates among all patients who attended Xuanwu Hospital in the six years between January 1, 2011 and December 31, 2016. Xuanwu hospital is a large general hospital located in downtown Beijing, China. The hospital’s electronic medical records (EMR) system served as the source for all data used in this study. The EMR system contains demographic information as well as all clinical information related to hospital department visits including HIV screening test dates and results. Information on diagnosis of HIV infection, including patients’ self-reported route of infection are also included.

### Eligibility

All individuals who attended Xuanwu Hospital between January 1, 2011 and December 31, 2016 were screened for inclusion in the study. Only those who received an HIV screening test dated between January 1, 2011 and December 31, 2016, and who had an HIV screening test result in their record were included in the study. Study sample size was not prospectively determined and no attempt was made to address potential sources of bias. Rather, all individuals meeting these criteria were included in the analysis.

### Testing and diagnosis

At Xuanwu Hospital, all HIV screening tests are performed in the same on-site hospital laboratory. Laboratory personnel were trained in HIV testing procedures according to National AIDS Testing Regulations[7], and were retrained on an annual basis. Laboratory audits were also regularly conducted to ensure compliance to approved procedures. Plasma samples were screened for HIV using the Architect HIV Ag/Ab Combo Reagent Kit (Abbott GmbH & Co. KG, Germany). All patients whose samples were non-reactive, had their results coded as “negative” in their EMR. All samples that yielded a reactive result were re-screened using the same procedure, on the same sample, in the same laboratory. All patients whose samples were non-reactive on re-screening, had their results coded as “negative” in their EMR, while all patients whose samples were reactive for a second time were coded as “possible positive.”

All outpatients with reactive HIV screening results (i.e. coded as “possible positive” for HIV in their EMR) were referred to HIV confirmatory testing at the Beijing Center for Disease Control and Prevention (CDC) laboratory. All inpatients’ samples were shipped to the Beijing CDC laboratory directly. Confirmatory HIV testing was performed by HIV-1 Western blot (WB) testing using the Western BloRing Kit (MP Biomedicals Asia Pacific, Singapore). For all patients with a positive WB result, a diagnosis of HIV infection was made, and their results were coded as “positive” in their EMR. If WB results were negative or inconclusive, patients’ EMRs continued to show a “possible positive” status and they were asked to return to repeat WB testing at a later date. All patients diagnosed with HIV infection were provided counseling and referred to treatment.

### Analysis

Categorical variables are presented as number and percent. Screening rate was calculated as the number of patients screened (numerator) divided by the total number of patients (denominator), expressed as a percentage. Detection rate was calculated as the number of confirmed cases of HIV infection (numerator) divided by the total number of patients screened (denominator), expressed per 10,000. Chi-square test and trend test were used to compare HIV detection rates over time. P-values <0.05 were considered statistically significant. All analyses were performed using SAS software (version 9.3, SAS Institute, USA).

### Ethics

This study was reviewed and approved by the Institutional Review Board of Xuanwu Hospital. Since HIV testing is routinely required before surgery, all inpatients requiring surgery were asked to provide informed consent for HIV testing and their information were depersonalized for research to understand and improve care services. Other inpatients who did not have surgery, and all outpatients signed written informed consent prior to HIV testing. All records, once extracted, were de-identified to ensure confidentiality.

## Results

As shown in **Table 1**, a total of 16,573,460 patients visited Xuanwu Hospital over the six years between January 1, 2011 and December 31, 2016. Among them, 235,961 patients received HIV serological screening tests, for an overall HIV screening rate of 1.4%. While a large majority of all patients who visited Xuanwu Hospital were outpatients (98.4%), most of those who received HIV screening were inpatients (70.0%), for an inpatient screening rate of 61.3% compared to an outpatient screening rate of 0.4%. Most patients screened attended the internal medicine (36.1%) and surgery (33.3%) departments. The highest screening rates were observed in the obstetrics and gynecology department (4.4%), followed by surgery (3.1%), eye, ear, nose, throat and mouth (1.4%), and internal medicine (1.0%) departments.

**Table 1:** HIV screening and detection rates at Xuanwu Hospital, Beijing, China, 2011–2016.

Characteristics	Patients Visited N (%)	Patients Screened N (%)	Screening Rate^a^ %	Patients Diagnosed N (%)	Detection Rate^b^, per 10,000
**Overall**	**16,573,460**	**235,961 (100)**	**1.4**	**140 (100)**	**5.93**
**Patient Status**					
Inpatient	269,799 (1.6)	165,276 (70.0)	61.3	74 (52.9)	4.48
Outpatient	16,303,661 (98.4)	70,685 (30.0)	0.4	66 (47.1)	9.34
**Department**					
Internal Medicine	8,887,892 (53.6)	85,290 (36.1)	1.0	47 (33.6)	5.51
Surgery	2,526,987 (15.2)	78,545 (33.3)	3.1	11 (7.9)	1.40
OB/Gyn	976,762 (5.9)	43,117 (18.3)	4.4	3 (2.1)	0.70
Eye, ENT, Mouth	1,210,762 (7.3)	17,237 (7.3)	1.4	4 (2.9)	2.32
Emergency	1,181,978 (7.1)	4,196 (1.8)	0.4	10 (7.1)	23.83
Dermatology/STI	573,766 (3.5)	4,160 (1.8)	0.7	64 (45.7)	153.85
Others	1,215,313 (7.3)	3,416 (1.4)	0.3	1 (0.7)	2.93

OB/Gyn: obstetrics and gynecology; ENT: ear, nose, and throat; STI: sexually transmitted infections

^a^Screening rate was calculated as the number of patients screened (numerator) divided by the number of patient visits (denominator), expressed as a percent

^b^Detection rate was calculated as the number of patients diagnosed (numerator) divided by the number of patients screened (denominator), expressed per 10,000

Among the 235,961 patients screened, a total of 140 patients were diagnosed with HIV infection, for an overall detection rate of 5.93 per 10,000. Highest detection rates were found among outpatients (9.34 per 10,000) and among patients attending the dermatology and STI department (153.85 per 10.000) and the emergency department (23.83 per 10,000; **Table 1**).

A stratification of detection rates by patient status and by year is presented in **Table 2**. Overall, the HIV detection rate was highest in 2011, at 13.22 per 10,000. In 2012, it dropped to 6.40 per 10,000, and then remained relatively stable, with no significant difference observed during the period from 2012 to 2016 (χ^2^ = 1.31, p = 0.86). A similar pattern was observed for inpatients—highest detection rate was observed in 2011 at 10.85 per 10,000, then in 2012 it dropped to 4.69 per 10,000, and no statistically significant difference detected in 2012 to 2016 (χ^2^ = 0.82, p = 0.93). For outpatients, detection rate was considerably higher in 2011 and 2012, at 22.43 per 10,000 and 11.19 per 10,000, respectively, after which the detection rate dropped to 7.96 per 10,000 and remained unchanged thereafter (χ^2^ = 2.28, p = 0.68).

**Table 2:** HIV cases and detection rates, stratified by year and by patient status, at Xuanwu Hospital, Beijing, China, 2011–2016.

	Overall			Outpatients			Inpatients		
Year	Screened N	Reactive N	Detection Rate^a^ per 10,000	Screened N (%)	Reactive N	Detection Rate^a^ per 10,000	Screened N (%)	Reactive N	Detection Rate^a^ per 10,000
**Overall**	**235,961**	**140**	**5.93**	**70,685 (0.43)**	**66**	**9.34**	**165,276 (61.26)**	**74**	**4.48**
2011	17,394	23	13.22	3,567 (0.16)	8	22.43	13,827 (34.94)	15	10.85
2012	37,529	24	6.40	9,834 (0.39)	11	11.19	27,695 (66.47)	13	4.69
2013	40,227	23	5.72	11,303 (0.41)	9	7.96	28,924 (65.46)	14	4.84
2014	46,024	23	5.00	16,508 (0.57)	12	7.27	29,516 (63.07)	11	3.73
2015	46,137	22	4.77	14,629 (0.49)	10	6.84	31,508 (66.25)	12	3.81
2016	48,650	25	5.14	14,844 (0.51)	9	6.06	33,806 (67.60)	16	4.73

^a^Detection rate was calculated as the number of patients diagnosed (numerator) divided by the number of patients screened (denominator), expressed per 10,000

A stratification of detection rates by hospital department and by year is presented in **Table 3**. Detection rate was highest in the dermatology and STI department in all years, despite a statistically significant decreasing trend over time (p = 0.004) from 362.32 per 10,000 in 2011 to 85.93 per 10,000 in 2016. The department with the next highest detection rates in most years was the emergency department. However, no statistically significant increasing trend over time was found (p = 0.20).

**Table 3:** HIV detection rates, stratified by department and by year, at Xuanwu Hospital, Beijing, China, 2011–2016.

	Detection Rate^a^ per 10,000					
Department	2011	2012	2013	2014	2015	2016
**Overall**	13.22	6.40	5.72	5.00	4.77	5.14
Internal Medicine	18.31	4.68	6.87	1.58	2.28	8.00
Surgery	3.36	1.63	1.50	0.69	1.96	0.58
OB/Gyn	0.00	1.28	1.15	1.40	0.00	0.00
Eye, ENT, Mouth	15.37	6.85	0.00	0.00	0.00	0.00
Emergency	25.00	12.58	30.53	0.00	34.80	36.86
Dermatology/STI	362.32	242.42	121.79	193.55	129.45	85.93
Others	69.93	0.00	0.00	0.00	0.00	0.00

OB/Gyn: obstetrics and gynecology; ENT: ear, nose, and throat; STI: sexually transmitted infection

^a^Detection rate was calculated as the number of patients diagnosed (numerator) divided by the number of patients screened (denominator), expressed per 10,000

As shown in **Table 4**, among the 140 patients diagnosed with HIV infection during the study period, most were 20 to 39 years of age (20–29: 40.0%, 30–39: 27.1%), and a large majority were male (91.4%). Most were born in mainland China, but outside Beijing (60.7%), unmarried (49.3%), and unemployed (55.0%). A majority reported having become infected via homosexual contact (70.0%).

**Table 4:** Characteristics of patients diagnosed with HIV infection, at Xuanwu Hospital, Beijing, China, 2011–2016.

Characteristics	Diagnosed N (%)
**Overall**	**140 (100)**
**Age, years**	
<20	2 (1.4)
20–29	56 (40.0)
30–39	38 (27.1)
40–49	21 (15.0)
50–59	14 (10.0)
≥60	9 (6.4)
**Sex**	
Male	128 (91.4)
Female	12 (8.6)
**Place of Birth**	
Beijing	48 (34.3)
Mainland China (not Beijing)	85 (60.7)
Hong Kong, Macao, Taiwan	7 (5.0)
**Education**	
<Middle school	11 (7.9)
Middle school	36 (25.7)
High school	36 (25.7)
≥College	57 (40.7)
**Marital Status**	
Unmarried	69 (49.3)
Married/cohabitating	55 (39.3)
Divorced/widowed	12 (8.6)
Missing	4 (2.9)
**Occupation**	
Student	6 (4.3)
Civil servant	14 (10.0)
Factory worker/farmer	14 (10.0)
Other	22 (15.7)
Retired	7 (5.0)
Unemployed	77 (55.0)
**Route of Infection**	
Homosexual contact	98 (70.0)
Heterosexual contact	40 (28.6)
Other	2 (1.4)

## Discussion

The main finding of our study was an overall HIV screening rate of 1.4% and HIV detection rate of 5.93 per 10,000 in Xuanwu Hospital during the six-year study period. Although screening rates increased after 2011, no statistically significant change was observed from 2012 to 2016. Interestingly, while screening rates were highest among inpatients and in the OB/Gyn and surgery departments, detection rates were highest among outpatients and in the dermatology and STI department and the emergency room. Finally, as expected, more than 98% of patients diagnosed with HIV during this period in Xuanwu Hospital reported sexual contact as their route of HIV infection, and most were young males who reported male-male sexual contact, reflecting not only the overall shift in China’s HIV epidemic toward sexual transmission[1], but also the rapid rise in HIV incidence and prevalence among MSM in China’s urban centers[1, 8].

We were not surprised to find substantially increased HIV screening rates at Xuanwu Hospital in 2012 (and thereafter), compared to 2011. In 2011, the Beijing Municipal Commission of the Health and Family Planning Commission issued a series of disease control working documents. These documents required that all Class 3/Grade A (3A) hospitals, like Xuanwu Hospital, must conduct more than 30,000 HIV screening tests each year[9]. We found that while there were just over 17 thousand screening tests performed in 2011, nearly 38 thousand were performed in 2012, a 2.2-fold increase. However, it is important to note that these documents also required that at least 80% of the HIV screening tests should be provided to outpatients in the dermatology and STI department, and at least 10% patients in the key departments including dermatology and STI, OB/gyn, urology/anorectal, infectious disease, and dialysis should be screened for HIV[9]. Results from our study show that neither of these requirements were met. Rather, our findings suggest that although the Beijing Municipal Commission’s disease control working documents were effective in increasing the volume of HIV screening, they were ineffective at ensuring HIV screening was targeted appropriately.

The overall HIV detection rate we observed at Xuanwu Hospital (2011–2016) of 5.93 per 10,000 was consistent with that reported at the first affiliated hospital of Nanjing Medical University (2010–2014)[10], but only roughly half that recently reported for another general hospital in Beijing, Peking Union Medical College Hospital (2003–2014). There, 715,421 patients were screened for HIV infection and 776 new cases were identified, for a detection rate of 10.85 per 10,000[11].

The HIV detection rate observed in the dermatology and STI department at Xuanwu Hospital stands out—it is 26 times higher than the detection rate for the hospital overall, and 6 times higher than the next highest department, emergency room. Though it is possible that health care providers in different departments may practice differently on providing HIV screening, for example, doctors in Dermatology/STI department may be more sensitive to the HIV related symptoms and risk sexual history, so that they can prescribe the test for people who are more likely to be positive. On the other hand, the highest HIV detection rate observed among patients attending Dermatology/STI department are most likely because of these patients are more likely having engaged in risk sexual behaviors compared to patients attending other department in the hospital.

Low HIV screening rate but high HIV detection rate departments may make patients miss opportunity of diagnose for those infected. During the study period, roughly 57 thousand outpatients with diagnosed STIs attended Xuanwu Hospital’s dermatology and STI department, yet only 0.7% received an HIV screening test (4160/573766 = 0.7%, Table 1). This seems startlingly low. Similarly, the department with the second highest detection rate at Xuanwu Hospital was the emergency room—four-fold higher than the hospital overall. Nevertheless, only 0.4% of emergency room visitors were screened for HIV. In a project conducted in six hospital emergency departments across Paris where routine HIV screening was implemented for one year, an overall screening rate of 3.9% resulted in 55 HIV cases being newly identified[12]. This model was associated with a moderately higher screening rate, yet a much higher detection rate than was found in our study.

Clearly, the screening rate at Xuanwu Hospital’s dermatology and STI department is not meeting the Beijing Municipal Commission’s guideline[9], and emergency is not listed as a key monitoring department required by the Beijing Municipal Health Authority[9]. Taken together with the observations made in the Peking Union Medical College Hospital study, where the top three hospital departments for HIV case identification were internal medicine (51%), emergency (18%), and dermatology and STI (14%)[11], our findings suggest an urgent need for, at a minimum, major revision of HIV screening guidelines for China’s hospital system. We strongly recommend that all visitors to hospital dermatology and STI departments be screened for HIV infection, that the emergency room should be added to the “key departments” for HIV screening, and that criteria be developed to help target an increased level of HIV screening in emergency rooms. Hospital emergency rooms should furthermore be included as key HIV monitoring settings, and equipped with rapid tests to ensure patients receive their results before leaving the ward.

Despite the dramatic scale up of HIV testing services, late presentation with HIV infection is a serious problem in China that causes excess morbidity and mortality and unnecessary onward transmission. A recent study by Cheng and colleagues in Guangzhou, China, has found that although the rate of late presentation is declining moderately, cases identified using the PITC strategy (like most in our study) had 37% greater odds of late presentation and 65% greater odds of presentation with advanced disease[13]. Therefore, to go one step further, we urge public health officials and policymakers to consider not only improved screening requirements for hospitals providing PITC, but also improvement of VCT services, and recognition, regulation, and promotion of HIV self-testing.

Our study had a number of important limitations. Firstly, due to the cross-sectional design, we were unable to investigate causality. Secondly, since our study population were seeking and receiving routine care, individual circumstances may have influenced HIV testing decisions by patients and/or providers. Thirdly, given very low prevalence and small number of patients being screening for HIV, the observed detection rates were unstable. For example, the detection rate in the emergency department in 2014 suddenly showed “0” while was in range from 12 to 36 per 10,000 patients in other years. It should be interpreted by chance, rather than any other reasons. Finally, the route of HIV infection variable relied on self-reporting by individuals after diagnosis and therefore exposures may be over- or under-estimated as a result of social-desirability or recall bias.

In conclusion, HIV screening in China’s general hospitals needs to be improved. More focus should be placed on screening outpatients, especially in the dermatology and STI department and in the emergency room. Moreover, special attention should be paid to screening young men. China still has a long way to go to reach the UNAIDS 90-90-90 Targets[4], and improving testing at its general hospitals is an important next step toward reaching these goals and to bringing its HIV epidemic under control. Public health officials and policymakers should urgently move to modify national guidelines for screening patients attending general hospitals for HIV. Moreover, they should consider improvements to other existing testing strategies, as well as promotion of HIV self-testing so that PLWH may be diagnosed earlier.

## Disclaimer

The views and opinions expressed herein belong to the authors alone, and do not represent the official policy, or endorsement of their affiliated institutions.

## Supporting information

S1 Checklist PLOSOne_Clinical_Studies_Checklist. (DOCX)

S2 Checklist STROBE_checklist_v4_combined_PlosMedicine. (DOCX)

S1 Certification IRB CERTIFICATION. (PDF)

S1 Data All data (Eng). (XLSX)
