# Supplementary material for: HIV screening among patients seeking care at Xuanwu Hospital: A cross-sectional study in Beijing, China, 2011–2016
Source: PLoS One. 2018 Dec 17;13(12):e0208008. doi: 10.1371/journal.pone.0208008 (PMC6296786; doi:10.1371/journal.pone.0208008)
Supplement: S2 Checklist — (DOCX) [file pone.0208008.s002.docx]

STROBE Statement—checklist of items for “**HIV screening among patients seeking care at Xuanwu Hospital: a cross-sectional study in Beijing, China, 2011–2016**”

|  | Item No. | Recommendation | Page  No. | Relevant text from manuscript |
| --- | --- | --- | --- | --- |
| **Title and abstract** | 1 | (*a*) Indicate the study’s design with a commonly used term in the title or the abstract | 1 | a cross-sectional study in Beijing, China, 2011–2016 |
|  |  | (*b*) Provide in the abstract an informative and balanced summary of what was done and what was found | 2 | “Although most were outpatients (98.4%), screening rate was higher among inpatients (70.0% versus 0.4%), and highest in internal medicine (36.1%) and surgery (33.3%) departments.” |
| Introduction | | | |  |
| Background/rationale | 2 | Explain the scientific background and rationale for the investigation being reported | 3 | “According to Chinese Government data, a total of 7.9 billion visits were made to all medical institutions nationwide in 2015, with a considerable proportion absorbed by general hospitals—2.3 billion outpatientsand123 million inpatients attended Chinese general hospitals in 2015[6].All general hospitals in China offer HIV screening, which visitors may access in three different ways.” |
| Objectives | 3 | State specific objectives, including any prespecified hypotheses | 3 | “we aimed to investigate HIV screening and detection rates (primary objective), and characteristics of patients diagnosed with HIV infection (secondary objective), at Xuan Wu Hospital,” |
| Methods | | | |  |
| Study design | 4 | Present key elements of study design early in the paper | 4 | “A cross-sectional study design was used to investigate HIV screening rates and HIV detection rates among all patients who attended Xuanwu Hospital in the six years between January 1, 2011 and December 31, 2016.” |
| Setting | 5 | Describe the setting, locations, and relevant dates, including periods of recruitment, exposure, follow-up, and data collection | 4 | “A cross-sectional study design was used to investigate HIV screening rates and HIV detection rates among all patients who attended Xuanwu Hospital in the six years between January 1, 2011 and December 31, 2016.  ”” Xuanwu hospital is a large general hospital located in downtown Beijing, China. The hospital’s electronic medical records (EMR) system served as the source for all data used in this study.” |
| Participants | 6 | (*a*) *Cohort study*—Give the eligibility criteria, and the sources and methods of selection of participants. Describe methods of follow-up  *Case-control study*—Give the eligibility criteria, and the sources and methods of case ascertainment and control selection. Give the rationale for the choice of cases and controls  *Cross-sectional study*—Give the eligibility criteria, and the sources and methods of selection of participants | 5 | “All individuals who attended Xuanwu Hospital between January 1, 2011 and December 31, 2016 were screened for inclusion in the study. Only those who received an HIV screening test dated between January 1, 2011 and December 31, 2016, and who had an HIV screening test result in their record were included in the study. Study size was not prospectively determined and no attempt was made to address potential sources of bias. Rather, all individuals meeting these criteria were included in the analysis.” |
|  |  | (*b*) *Cohort study*—For matched studies, give matching criteria and number of exposed and unexposed  *Case-control study*—For matched studies, give matching criteria and the number of controls per case |  |  |
| Variables | 7 | Clearly define all outcomes, exposures, predictors, potential confounders, and effect modifiers. Give diagnostic criteria, if applicable | 6 | “screening rate”” Detection rate” |
| Data sources/ measurement | 8* | For each variable of interest, give sources of data and details of methods of assessment (measurement). Describe comparability of assessment methods if there is more than one group | *6* | Screening rate was calculated as the number of patients screened (numerator) divided by the total number of patients (denominator), expressed as a percentage. Detection rate was calculated as the number of confirmed cases of HIV infection (numerator) divided by the total number of patients screened (denominator), expressed per 10,000.Chi-square test and trend test were used to compare HIV detection rates over time. P-values <0.05 were considered statistically significant. All analyses were performed using SAS software (version 9.3, SAS Institute, USA). |
| Bias | 9 | Describe any efforts to address potential sources of bias | 6 | “Study size was not prospectively determined and no attempt was made to address potential sources of bias. Rather, all individuals meeting these criteria were included in the analysis.” |
| Study size | 10 | Explain how the study size was arrived at | 5 | “All individuals who attended Xuanwu Hospital between January 1, 2011 and December 31, 2016 were screened for inclusion in the study. Only those who received an HIV screening test dated between January 1, 2011 and December 31, 2016, and who had an HIV screening test result in their record were included in the study. Study size was not prospectively determined and no attempt was made to address potential sources of bias. Rather, all individuals meeting these criteria were included in the analysis.” |

Continued on next page

| Quantitative variables | 11 | Explain how quantitative variables were handled in the analyses. If applicable, describe which groupings were chosen and why | 6 | Screening rate was calculated as the number of patients screened (numerator) divided by the total number of patients (denominator), expressed as a percentage. Detection rate was calculated as the number of confirmed cases of HIV infection (numerator) divided by the total number of patients screened (denominator), expressed per 10,000.” |
| --- | --- | --- | --- | --- |
| Statistical methods | 12 | (*a*) Describe all statistical methods, including those used to control for confounding | 6 | “Chi-square test and trend test were used to compare HIV detection rates over time. P-values <0.05 were considered statistically significant. All analyses were performed using SAS software (version 9.3, SAS Institute, USA).” |
|  |  | (*b*) Describe any methods used to examine subgroups and interactions | 6 | “Categorical variables like number of patients in different clinical divisions are presented as number and percent.” |
|  |  | (*c*) Explain how missing data were addressed | 7 | No missing data in EMS system. |
|  |  | (*d*) *Cohort study*—If applicable, explain how loss to follow-up was addressed  *Case-control study*—If applicable, explain how matching of cases and controls was addressed  *Cross-sectional study*—If applicable, describe analytical methods taking account of sampling strategy | 5 | No sampling. “Study size was not prospectively determined and no attempt was made to address potential sources of bias. Rather, all individuals meeting these criteria were included in the analysis.” |
|  |  | (*e*) Describe any sensitivity analyses |  | No |
| Results | | | | |
| Participants | 13* | (a) Report numbers of individuals at each stage of study—eg numbers potentially eligible, examined for eligibility, confirmed eligible, included in the study, completing follow-up, and analysed | 5 | No information like this. Because” “All individuals who attended Xuanwu Hospital between January 1, 2011 and December 31, 2016 were screened for inclusion in the study. Only those who received an HIV screening test dated between January 1, 2011 and December 31, 2016, and who had an HIV screening test result in their record were included in the study.” |
|  |  | (b) Give reasons for non-participation at each stage | 5 | No information like this. Because” “All individuals who attended Xuanwu Hospital between January 1, 2011 and December 31, 2016 were screened for inclusion in the study. Only those who received an HIV screening test dated between January 1, 2011 and December 31, 2016, and who had an HIV screening test result in their record were included in the study.” |
|  |  | (c) Consider use of a flow diagram |  | Not necessary in this paper. |
| Descriptive data | 14* | (a) Give characteristics of study participants (eg demographic, clinical, social) and information on exposures and potential confounders | 7 | While a large majority of all patients who visited Xuanwu Hospital were outpatients (98.4%), most of those who received HIV screening were inpatients (70.0%), |
|  |  | (b) Indicate number of participants with missing data for each variable of interest |  | No missing data |
|  |  | (c) *Cohort study*—Summarise follow-up time (eg, average and total amount) |  |  |
| Outcome data | 15* | *Cohort study*—Report numbers of outcome events or summary measures over time |  |  |
|  |  | *Case-control study—*Report numbers in each exposure category, or summary measures of exposure |  |  |
|  |  | *Cross-sectional study—*Report numbers of outcome events or summary measures | *8* | “a total of 16,573,460 patients visited Xuanwu Hospital over the six years between January 1, 2011 and December 31, 2016. No missing data in EMS system. Among them, 235,961 patients received HIV serological screening tests, for an overall HIV screening rate of 1.4%.” |
| Main results | 16 | (*a*) Give unadjusted estimates and, if applicable, confounder-adjusted estimates and their precision (eg, 95% confidence interval). Make clear which confounders were adjusted for and why they were included |  | Not necessary in this paper |
|  |  | (*b*) Report category boundaries when continuous variables were categorized |  | See table 1, 2, and 3. Only categories of clinical division, years, and outpatients and inpatients were used. |
|  |  | (*c*) If relevant, consider translating estimates of relative risk into absolute risk for a meaningful time period |  | Not necessary in this paper |

Continued on next page

| Other analyses | 17 | Report other analyses done—eg analyses of subgroups and interactions, and sensitivity analyses |  | Not necessary in this paper. |
| --- | --- | --- | --- | --- |
| Discussion | | | | |
| Key results | 18 | Summarise key results with reference to study objectives | 8 | “The main finding of our study was an overall HIV screening rate of 1.4% and HIV detection rate of 5.93 per 10,000 in Xuanwu Hospital during the six-year study period.” |
| Limitations | 19 | Discuss limitations of the study, taking into account sources of potential bias or imprecision. Discuss both direction and magnitude of any potential bias | 11 | “Our study had a number of important limitations.” |
| Interpretation | 20 | Give a cautious overall interpretation of results considering objectives, limitations, multiplicity of analyses, results from similar studies, and other relevant evidence | 8-10 | “In a project conducted in six hospital emergency departments across Paris where routine HIV screening was implemented for one year, an overall screening rate of 3.9% resulted in 55 HIV cases being newly identified” |
| Generalisability | 21 | Discuss the generalisability (external validity) of the study results | 11 | In conclusion, HIV screening in China’s general hospitals needs to be improved. More focus should be placed on screening outpatients, especially in the dermatology and STI department and in the emergency room. |
| Other information | |  | | |
| Funding | 22 | Give the source of funding and the role of the funders for the present study and, if applicable, for the original study on which the present article is based | 12 | “This work was supported by the National Health and Family Planning Commission of the People’s Republic of China ( #131-13-000 -105-01 and # 2012ZX10001-007).” |

*Give information separately for cases and controls in case-control studies and, if applicable, for exposed and unexposed groups in cohort and cross-sectional studies.

**Note:** An Explanation and Elaboration article discusses each checklist item and gives methodological background and published examples of transparent reporting. The STROBE checklist is best used in conjunction with this article (freely available on the Web sites of PLoS Medicine at http://www.plosmedicine.org/, Annals of Internal Medicine at http://www.annals.org/, and Epidemiology at http://www.epidem.com/). Information on the STROBE Initiative is available at www.strobe-statement.org.
