# Supplementary material for: HIV screening among patients seeking care at Xuanwu Hospital: A cross-sectional study in Beijing, China, 2011–2016
Source: PLoS One. 2018 Dec 17;13(12):e0208008. doi: 10.1371/journal.pone.0208008 (PMC6296786; doi:10.1371/journal.pone.0208008)
Supplement: S1 Certification — (PDF) [file pone.0208008.s003.pdf]

To PI

Ethics committee of Xuanwu Hospital, Capital Medical University

Approval Letter about the Ethical Review of Clinical Scientific Research

Number of Approval Letter: 临研文审[2018]001 号

**Title of the Clinical Research**	HIV screening results among patients seeking medical care at Xuanwu Hospital: a cross-sectional study in Beijing, China, 2011–2016		
**Project Source**	None		
**Project Number**	NA		
**Hosted by**	Information Center, Xuanwu Hospital, Capital Medical University	**Primary Investigator**	Rui Li
**Department Head for hosting the research**	Dr. Zhi-gang Liang		
**Review approaches**	Review at<br>☐Full Board Meeting<br>■Expedited Review	**Date of Review**	January 25th ,2018
**Will the research process be subject to the continuous review by the Ethics Committee?**<br>■Yes ☐No			
**From the date on which the research is approved, the review frequency:**<br>☐3 months ☐6 months ■12 months ☐other:________			
**Period of Validity for the Approval Letter**	From January 25th ,2018 to January 25th ,2019		
